# Adaptive frequency-based modeling of whole-brain oscillations: Predicting regional vulnerability and hazardousness rates

**DOI:** 10.1162/netn_a_00104

**Published:** 2019-09-01

**Authors:** Neda Kaboodvand, Martijn P. van den Heuvel, Peter Fransson

**Affiliations:** Department of Clinical Neuroscience, Karolinska Institutet, Stockholm, Sweden; Dutch Connectome Lab, Department of Complex Traits Genetics, Center for Neurogenomics and Cognitive Research, VU Amsterdam, Amsterdam, The Netherlands; Department of Clinical Genetics, VU University Medical Center, Amsterdam Neuroscience, Amsterdam, 1081 HV, The Netherlands; Department of Clinical Neuroscience, Karolinska Institutet, Stockholm, Sweden

**Keywords:** Resting-state fMRI, Whole-brain network modeling, Dynamical systems, Adaptive frequency, In silico perturbation, Vulnerability

## Abstract

Whole-brain computational modeling based on structural connectivity has shown great promise in successfully simulating fMRI BOLD signals with temporal coactivation patterns that are highly similar to empirical functional connectivity patterns during resting state. Importantly, previous studies have shown that spontaneous fluctuations in coactivation patterns of distributed brain regions have an inherent dynamic nature with regard to the frequency spectrum of intrinsic brain oscillations. In this modeling study, we introduced frequency dynamics into a system of coupled oscillators, where each oscillator represents the local mean-field model of a brain region. We first showed that the collective behavior of interacting oscillators reproduces previously shown features of brain dynamics. Second, we examined the effect of simulated lesions in gray matter by applying an in silico perturbation protocol to the brain model. We present a new approach to map the effects of vulnerability in brain networks and introduce a measure of regional hazardousness based on mapping of the degree of divergence in a feature space.

## Author Summary

Computational modeling of the brain enables us to test different hypotheses without any experimental complication, and it provides us with a platform for improving our understanding of different brain mechanisms. In this study, we proposed a new macroscopic computational model of the brain oscillations for resting-state fMRI. Optimizing model parameters using empirical data was performed based on several measures of functional connectivity and instantaneous coherence. We simulated the effect of malfunction in a brain region by changing that region’s dynamics to evoke noisy behavior. Together with presenting a new paradigm for local vulnerability mapping in the brain connectome, we evaluated the hazard rate induced after perturbing a brain region by measuring divergence of the perturbed model from the original model in feature space. The analysis of hazard rates induced by primary failures of individual brain regions provides relevant insights not only into the size of the damage inflicted on the connectome by a particular failure, but also into the potential origins of disease. Furthermore, we proposed a spatial brain map that is associated with the regional hazardousness rates, which is in good agreement with the known pathophysiologic roles of malfunction in different functional systems in the brain.

## INTRODUCTION

The human connectome is a complex network that is made up of interactive systems encompassing a large number of regions. Regions communicate with each other in order to share and process information providing the structural and functional basis for complex cognitive processes. Understanding the network architecture of the human brain in the context of its robustness and the integration between different subnetworks (known as functional systems) has received increased attention in system-level network neuroscience. However, we still have limited knowledge of the emergence of brain dynamics from the underlying anatomy. So far, there is evidence suggesting that the large-scale structural connectome of the brain constrains the strength and persistence of resting-state functional connectivity (FC; Honey et al., 2009), with significant contributions of structural connections for the integrated state (Fukushima, Betzel, He, van den Heuvel, et al., 2018). An impaired structural connectome may lead to disrupted FC, which contributes to neurodegenerative diseases (for example, see Griffa, Baumann, Thiran, & Hagmann, 2013). The interplay between the brain’s structure and dynamics underlies all brain functions spanning from consciousness and perception to learning, memory, and movement (Deco, Jirsa, Robinson, Breakspear, & Friston, 2008). Moreover, the relationship between the structural backbone and the dynamics of brain activity is believed to play an important role for surviving network communication failures and attacks (Barabási, 2016). In the past decade we have witnessed great progress towards the systematic modeling of the neural network dynamics (Breakspear, Heitmann, & Daffertshofer, 2010; Cabral, Kringelbach, & Deco, 2014; Fink, 2018). Large-scale computational models are uniquely suited to address difficult questions related to the role of the brain’s structural network in shaping measures of FC averaged across longer timescales (Cabral et al., 2014; Ritter, Schirner, McIntosh, & Jirsa, 2013). Additionally, we can study the emergence of complex brain dynamics (Roberts et al., 2018), through time-varying analyses of functional coherence.

Several resting-state studies have shown that there exist spontaneous fluctuations in coactivation patterns of distributed brain regions (Hutchison et al., 2013; Thompson, Brantefors, & Fransson, 2017; Zalesky, Fornito, Cocchi, Gollo, & Breakspear, 2014), which gives rise to an efficient information exchange while minimizing metabolic expenditure (Zalesky et al., 2014). Furthermore, spectral analysis of brain fluctuations has disclosed valuable information about the underlying sources of time-varying connectivity of brain regions (C. Chang & Glover, 2010; Ries et al., 2018; Yaesoubi, Allen, Miller, & Calhoun, 2015). These studies suggest that the frequency spectrum of intrinsic oscillations of the brain has a time-varying nature.

In this study, we present a theoretical framework for modeling large-scale brain dynamics based on the theory of dynamical systems. Dynamical systems theory is a mathematical framework used to describe the behavior of the complex dynamical systems (i.e., systems that evolve in time), usually by a set of differential equations (Strogatz, 2018). The proposed model is used to show that important questions related to the prediction of perturbation patterns can be tackled in an accessible and useful way. Furthermore, we show that our model can be employed to simulate perturbations in different brain regions to assess the vulnerability and hazardousness of individual connections and brain regions.

To do this, we start by constructing a macroscopic computational model of the brain where local brain regions, each modeled by a local mean-field model, are interacting through the structural brain network architecture (Breakspear, 2017; Cabral et al., 2014; Deco & Jirsa, 2012; Deco, Jirsa, McIntosh, Sporns, & Kötter, 2009; Gollo, Zalesky, Hutchison, van den Heuvel, & Breakspear, 2015; Honey, Kötter, Breakspear, & Sporns, 2007; Honey et al., 2009). We then suggest that the local dynamics of each brain area can be described by a modified Stuart-Landau equation. Subsequently, we show that simulated BOLD signals are forming FC patterns that are similar to the FC patterns obtained from measured BOLD signals. This similarity was found to be valid both in terms of strength of FC and in the form of the establishment of communities of brain regions as shown in previous resting-state network studies (Yeo et al., 2011). Additionally, we show that temporal structure of simulated BOLD signals is highly similar to the fluctuations of empirical BOLD signals.

Of note, by perturbing different brain regions in our model and measuring how the system responds to the induced failures, we can obtain information on the underlying association between structure and dynamics in the brain. Previous studies have simulated the effects of brain lesions on both local and global levels of network activity, for example by removing individual connections (edges) or all connections of an individual region from the network (Aerts, Fias, Caeyenberghs, & Marinazzo, 2016; Cabral, Hugues, Kringelbach, & Deco, 2012; Deco, Van Hartevelt, Fernandes, Stevner, & Kringelbach, 2017; Váša et al., 2015). However, applying models with Hopf bifurcation are shown to be particularly well suited for in silico perturbation studies (Deco et al., 2018; Saenger et al., 2017). Therefore, in this study we used an in silico perturbation protocol by applying bifurcation-induced shifts in the dynamical regime of each individual brain region. Regime refers to the characteristic behavior of a dynamical system, and regime shifts are sudden, large, and persistent changes in the function of the system as a result of some external source of disturbance (Folke et al., 2004; Holling, 1973; Scheffer, Carpenter, Foley, Folke, & Walker, 2001). Static as well as dynamic measures of FC patterns were investigated for different in silico failures. This analysis was followed up by applying the representational similarity analysis (RSA) framework to investigate how a targeted brain region’s failure contributed to an increased distance between the perturbed connectome versus the healthy connectome in RSA space. Further, we suggest that the aforementioned distance in turn provides useful information about the degree of regional hazardousness. Moreover, we propose that our approach to modeling brain dynamics is helpful to understand the diversity of fragility for brain regions in the connectome with regard to injuries and disease. We suggest that investigating perturbation maps that are aggregated from different types of perturbation targets is a useful marker to estimate the degree of individual regions’ and/or connections’ vulnerability in the brain connectome.

Quantification of perturbation patterns provided by dynamical systems modeling of the brain may become a helpful tool when designing goal-directed interventions such as presenting sensory stimuli and interventions like applying transcranial magnetic stimulation. Furthermore, given the promising results of new closed-loop deep brain stimulations (DBS) that are based on ongoing brain activity (Weerasinghe et al., 2018), we believe that a mathematical model of the brain oscillations will be helpful in designing an optimal stimulation strategy that provides detailed information about the particular state of the system that is requited. It may also allow us to better understand why some brain lesions cause cognitive and physical impairment that may become more severe over time while other lesion patterns have a much a better long-term outcome.

## MATERIALS AND METHODS

### Data Used and Preprocessing

The primary data source for this study was the Human Connectome 500-subject release (Smith et al., 2013; Van Essen et al., 2012). Subject recruitment procedures and informed-consent forms were approved by the Washington University Institutional Review Board. The dataset is publicly shared on the ConnectomeDB database (https://db.humanconnectome.org). Resting-state fMRI data were collected in two sessions, each session including two runs with phase encoding in either left-to-right or right-to-left directions. For our analysis, we used a single resting-state run, collected during 14.4 min with temporal resolution of 0.72 s. The entire dataset consisted of 1,200 image volumes.

The dataset had been minimally preprocessed (Glasser et al., 2013; Smith et al., 2013; Van Essen et al., 2012), which starts with gradient distortion correction and proceeds by realignment, bias field correction, spatial distortion removal, registration to standard Montreal Neurological Institute (MNI) space, and intensity normalization (Glasser et al., 2013). Also, ICA +FIX (“FMRIB’s ICA-based X-noiseifier”) pipeline had been applied in order to automatically remove nuisance components (e.g., motion effects, nonneuronal physiology, and scanner artefacts) from the fMRI data (Griffanti et al., 2014; Salimi-Khorshidi et al., 2014). Further preprocessing steps were added to the pipeline. The volumes collected during the first 10 s of the scan as well as the outlier volumes were discarded. Using the 3dDespike function in AFNI, outlier volumes were detected and interpolated from neighboring volumes. Next, the nuisance regression was performed using the global signal, mean white matter and cerebrospinal fluid (CSF) signals, as well as the 24 motion time series (C.G. Yan, Craddock, Zuo, Zang, & Milham, 2013) simultaneously with linear and quadratic detrending. In addition, the data were bandpass filtered (0.02–0.12 Hz; Fukushima, Betzel, He, de Reus, et al., 2018). Moreover, the FreeSurfer software (https://surfer.nmr.mgh.harvard.edu) was applied to parcellate the cortical surface of T1-weighted images into 68 anatomically segregated gyral-based regions of interest (Desikan et al., 2006). This data-driven parcellation is also known as the Desikan-Killiany cortical atlas.

High-quality diffusion-weighted MRI data for the same 500 subjects from the HCP consortium (Glasser et al., 2013; Van Essen et al., 2012) was used for a streamline tractography on 68 cortical regions (Yeh, Wedeen, & Tseng, 2010). Next, we created subject-level weighted structural connectomes using measures of streamline density, computed by dividing the number of streamlines connecting two regions by the average of the volumes of the two interconnected regions to obtain streamline density (Hagmann et al., 2008; van den Heuvel, Kahn, Goñi, & Sporns, 2012; van den Heuvel & Sporns, 2011). For details on the processing steps of diffusion MRI-derived connectivity data we refer the reader to the earlier work (van den Heuvel & Sporns, 2011). We constructed a group-representative structural connectome by averaging the subject-level structural connectivity entries that had nonzero values for at least 60% of the subjects (de Reus & van den Heuvel, 2013), followed by resampling the data to follow a Gaussian distribution with *μ* = 0.5 and *σ* = 0.15 (Honey et al., 2009; van den Heuvel et al., 2015).

### Computational Modeling of Brain Dynamics

In the system-level model of the brain, each region can be modeled by a local mean-field model, and they interact with each other through the structural connectome as previously described (Breakspear, 2017; Cabral et al., 2014; Deco & Jirsa, 2012; Deco et al., 2009; Gollo et al., 2015; Honey et al., 2007; Honey et al., 2009). Accordingly, we model the local dynamics of each brain area with a modified Stuart-Landau equation. The Stuart-Landau equation describes the behavior of a nonlinear oscillating system near the Hopf bifurcation, and it can be thought of as the principal model for nonlinear oscillators since it is the simplest possible model to describe amplitude dynamics (Röhm, Lüdge, & Schneider, 2018). When coupled together, the collective behavior of interacting oscillator systems has been shown to reproduce features of brain dynamics (Deco, Kringelbach, Jirsa, & Ritter, 2017; Freyer et al., 2011). Graph theory allows representing the interactions within the resulting complex network through a set of nodes that are connected by edges (Newman, 2003; Rubinov & Sporns, 2010; Strogatz, 2001). Since here we are modeling the neural activity of each brain region with the oscillations produced by an oscillator’s model, the words “brain region,” “node,” and “oscillator” will be used interchangeably in the text.

#### Local mean-field model.

The Stuart-Landau equation describes the dynamic behavior of each oscillator *j* by

$${\dot{\text{z}}}_{\text{j}}=\left(\text{a}+\text{i}\omega_{\text{j}}-{\left|{\text{z}}_{\text{j}}\right|}^{\text{2}}\right){\text{z}}_{\text{j}},$$ (1)

where *z* = *r* *e*^*iθ*^ = *r* cos *θ* + *ir* sin *θ* is a complex number describing the state of the oscillator, ω ∈ ℝ is the frequency of each oscillator, and the bifurcation parameter a ∈ ℝ determines whether the oscillator is characterized by noisy fluctuations or exhibits oscillatory behavior.

By applying a slight change to the control parameter of the nonlinear dynamical system, an abrupt qualitative change in the behavior of the system may occur, which is called bifurcation. For example, if the control parameter (a) in Equation 1 moves from the negative to the positive domain, a critical point is reached when the pair of complex eigenvalues for the oscillator crosses the imaginary axis. At this point, the equilibrium of the system loses its stability and a stable isolated periodic trajectory (known as limit cycle) of radius $\sqrt{a}$ with a constant angular frequency *ω* develops. This phenomenon is called a supercritical Hopf bifurcation (Supplementary Figure 1). The closed trajectory in the phase-space represents periodic behavior of the system (Hilborn, 2000; Kuramoto, 1984; Strogatz, 2018).

Nonlinear dynamic models with stable limit cycles describe systems with self-sustained oscillations (i.e., oscillating behavior persists even in the absence of external periodic forcing or facing with slight perturbations). The nonlinear system described above is easier to analyze if we rewrite Equation 1 in polar coordinates, which in turn give us the equations below:

$$\overset{z=re^{i\theta }}{\Rightarrow }\left\{\begin{array}{c} {\dot{\text{r}}}_{\text{j}}=\text{a}r_{j}-r_{j}^{3}\\ {\dot{\theta }}_{\text{j}}=\omega_{\text{j}} \end{array}\right..$$ (2)

If we translate the model stated in Equation 2 to functional neuroimaging data, we can interpret the real part of the variable *z* (i.e., *r* cos *θ*) as an indirect measure of brain activity acquired by the MR scanner, whereas the imaginary part serves as the hidden state of the system that is unobservable.

#### Modeling whole-brain dynamics—Collective neurodynamics.

The next step is to embed the local dynamics for each node as postulated in Equations 1 and 2 into a large-scale model that encapsulates all nodes in the brain connectome. Then, the dynamics of the whole brain is described by a system of coupled differential equations as given below:

$${\dot{\text{z}}}_{\text{j}}=\left(\text{a}+\text{i}\omega_{\text{j}}-{\left|{\text{z}}_{\text{j}}\right|}^{\text{2}}\right){\text{z}}_{\text{j}}+\text{G}\sum {\text{C}}_{\text{ij}}\left({\text{z}}_{\text{i}}-{\text{z}}_{\text{j}}\right)+\beta \eta_{\text{j}}.$$ (3)

In Equation 3, G ∈ ℝ is the global coupling parameter where the strength of coupling between regions is set by the structural connectivity matrix C (Arenas, Díaz-Guilera, Kurths, Moreno, & Zhou, 2008; Deco, Kringelbach et al., 2017; Rodrigues, Peron, Ji, & Kurths, 2016). The constant G serves as a common tuning parameter that scales all the connection weights similarly.

In addition, additive Gaussian noise (denoted with η_j_ with standard deviation of *β* = 0.02 and implemented as Wiener process) was added to the differential equation of each oscillator to simulate the effect of random processes that occur in brain (e.g., stochastic effects of ion channels and heat), as well as inputs from sensory systems that have not been explicitly modeled (Roberts, Friston, & Breakspear, 2017).

#### Introducing frequency dynamics (free-running frequency evolution).

We describe the dynamics of angular frequency for every area *j* as

$${\dot{\omega }}_{\text{j}}={\omega^{0}}_{\text{j}}-\lambda \;\omega_{\text{j}},$$ (4)

where we introduce the coefficient λ ∈ ℝ as the frequency lethargy. For brain region *j*, the angular frequency *ω*_*j*_ represents the free-running frequency, and ω^0^_j_ is its intrinsic frequency. The intrinsic frequency for brain region *j* was estimated from the actual BOLD signals of that particular region, as given by the median (across subjects) peak frequency of the mean (across voxels) BOLD signal.

Notably, in this study we introduce the possibility of frequency modulation of each oscillator, where the oscillating frequency can be modulated by its neighbors (we will later show that the frequency change for each region can be modulated by the net phase of all oscillators in its direct neighborhood). Thus, by combining the previous model of whole-brain dynamics (as formulated in Equation 3) with the suggested frequency modulation equation, we arrive at the following coupled differential equations to describe the whole-brain dynamics in the connectome:

$$\left\{\begin{array}{c} {\dot{\text{z}}}_{\text{j}}=\left(\text{a}+\text{i}\omega_{\text{j}}-{\left|{\text{z}}_{\text{j}}\right|}^{\text{2}}\right){\text{z}}_{\text{j}}+\text{G}\sum {\text{C}}_{\text{ij}}\left({\text{z}}_{\text{i}}-{\text{z}}_{\text{j}}\right)+\beta \eta_{\text{j}}\\ {\dot{\omega }}_{\text{j}}={\omega^{0}}_{\text{j}}-\lambda \;\omega_{\text{j}}+m\;\psi_{\text{j}} \end{array}\right..$$ (5)

The global phase of the neighbor ensemble for oscillator *j* is denoted by phase angle *ψ*_j_, which represents the interacting phases of the ensemble of all oscillators connected to this particular oscillator. Additionally, the model stated in Equation 5 includes the frequency modulation coefficient *m*, which needs to be optimized for the BOLD data. The frequency modulation term was introduced to test the hypothesis that it could offer a suitable platform to understand the dynamic organization of synchronization patterns in the brain connectome.

**Figure 1.**
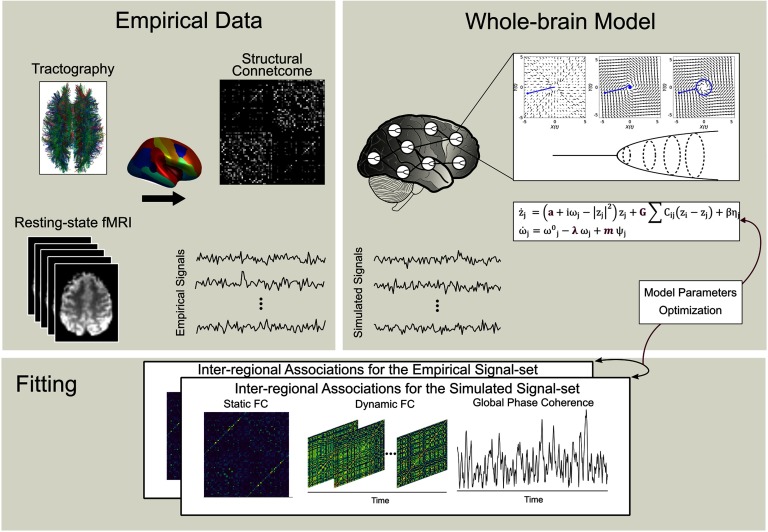
Construction of whole-brain network model. Using diffusion-MRI, streamline tractography was performed on 68 cortical regions. Subsequently, a group-representative structural connectome was built to govern the interaction strength between different regions. Resting-state brain dynamics was simulated using our adaptive frequency-based model. The model parameter set was tuned using the empirical data, based on several measures of functional connectivity and instantaneous coherence.

In our model, frequency modulation is achieved through the structural connectivity matrix so that for each oscillator it limits the inclusion of oscillators in the neighbor ensemble to the ones that are directly structurally connected to that particular oscillator (ψ_j_ = ∑ C_ij_*θ*_*i*_). The phase of each oscillator (denoted by *θ*_*i*_) was calculated as the inverse tangent ([−*π*/2, *π*/2]) of the quotient of dividing the imaginary part by the real part of the system variable (*z*_*i*_). Schematic overview of whole-brain modeling is illustrated in Figure 1.

### Identification of the Optimal Working Point for the Model

The global coupling parameter *G*, the global bifurcation parameter *a*, the frequency lethargy coefficient *λ*, and the frequency modulation coefficient *m* are the parameters of our model, which are required to be optimized in order to find a working point where the simulated signals maximally fit the empirical BOLD signals. We performed a grid-search framework to estimate optimal values for model parameters. Maximum fitness was achieved by minimizing the dissimilarity between model-driven measures and the metrics extracted based on the empirical BOLD signals. Measures of interest included time-averaged measures such as the Pearson correlation of static FC patterns and modularity that quantifies the level of segregation for the resting-state subnetworks, as well as three measures calculated based on instantaneous phase of signals. We computed a composite score by converting each metric to a unity–based normalized distance between model and empirical data and subsequently averaging them (Supplementary Equations). Applied methods for computation of these measures are summarized below.

#### Measures based on time-averaged functional connectivity.

##### Static functional connectivity patterns

The matrix of correlation coefficients was computed from the preprocessed BOLD signals originating from all 68 regions of interest as included in the Desikan-Killiany cortical atlas. A group-level FC matrix was calculated by averaging the z-transformed subject-level connectivity matrices. The Pearson correlation coefficient between the group-level FC matrix and the FC matrix obtained for the simulated signals was used as one of the measures, indicative of similarity between empirical and simulated BOLD signals.

##### Whole-brain modularity

Previous research has found functional networks/systems as ensembles of brain regions that coactivate during the resting state as well as during tasks (Smith et al., 2009). In order to associate every region of brain with a functional network, we first overlaid the 68 regions with the functional networks (so-called resting-state networks) that were previously defined based on the similarity of intrinsic FC profiles in 1,000 subjects (Yeo et al., 2011). Based on the percentage of overlap, we related each region with one of the functional networks. Accordingly, the six functional networks considered here included the default mode (DM), the limbic (LIM), the dorsal attention or control (dTT/CONT), the salience or ventral attention (SAL/vATT), the somatomotor (SOM), and the visual (VIS) networks. Supplementary Figure 2 displays the layout of these functional networks. The abbreviations used for the brain regions are shown in Supplementary Table 1.

Indeed, the whole-brain network can be divided into several modules (Betzel et al., 2014; Rubinov & Sporns, 2011) that are in good agreement with known functional systems (Meunier, Lambiotte, Fornito, Ersche, & Bullmore, 2009; Power et al., 2011). The presence of these modules that correspond to the functional systems is an indication that the simulated network adequately models some characteristics of brain organization.

Modularity quantifies the degree to which a network can be divided into the groups of nodes (i.e., modules) with stronger intramodule connections and weaker intermodule connections. For an FC matrix with positive weights, modularity is defined as

$$Q=\frac{1}{v}\underset{i,j}{\sum }(w_{ij}-\frac{s_{i}s_{j}}{v})\delta_{ij},$$ (6)

where the *i*, *j*-th element of the FC matrix is denoted by *w*_*ij*_, and *s*_*i*_ = ∑_*j*_ *w*_*ij*_ is the nodal strength. The variable *v* = ∑_*ij*_ *w*_*ij*_ is the overall weight of the network. The Kronecker delta function *δ*_*ij*_ is equal to 1 if the *i*-th and *j*-th nodes belong to the same module, and 0 otherwise. Hence, we computed the modularity (*Q*) of whole-brain network, assuming the abovementioned functional networks (DM, LIM, dATT/CONT, SAL/vATT, SOM, and VIS) are the brain network’s modules.

#### Instantaneous phase-based measures.

Here, we applied the Hilbert transformation to the regional BOLD signals to derive the analytic representation of the real-valued BOLD signals. We calculated the instantaneous phase of the analytic signal by computing the four-quadrant inverse tangent (*tan*^−1^) of the quotient formed by dividing the imaginary part by the real part of the BOLD signal.

##### Dynamic functional connectivity patterns

Computing the instantaneous phase synchrony (phase coherence) as a measure of time-varying FC offers single time point resolution and has gained considerable attention in the recent literature (Omidvarnia et al., 2016; Pedersen, Omidvarnia, Walz, Zalesky, & Jackson, 2017; Ponce-Alvarez et al., 2015). The instantaneous FC for each pair of regions was defined by cosine similarity of the phases obtained from associated regions’ signals. Thus, it was computed as 1 − |sin(Δθ)| at each time point, where Δθ represents the instantaneous phase difference between two BOLD signals.

Next, the similarity between instantaneous FC measures of different time points was calculated based on the cosine similarity between vectors created by applying half-vectorization on every instantaneous FC matrix. Next, we selected the cumulative distribution of the concatenated cosine similarities across all subjects as a measure of dynamic connectivity (Cabral, Kringelbach, & Deco, 2017; Deco & Kringelbach, 2016; Deco, Kringelbach et al., 2017; Senden, Reuter, van den Heuvel, Goebel, & Deco, 2017). The same procedure was applied to the simulated data.

As a final step, we applied the Kolmogorov-Smirnoff test to evaluate the degree of agreement between dynamic FC patterns obtained by the empirical BOLD data and the results generated by our model. This comparison was performed for all tested combinations of the model parameters.

##### Macroscopic coherence of the model system

At the edge of the critical point of the bifurcation in a system of coupled oscillators, there is a transition of the global attractor from incoherence to synchrony (Skardal, Ott, & Restrepo, 2011), which can be defined through the emergence of a macroscopic mean-field. This mean-field is computed as the centroid vector of the phase distribution as

$$R=r\;exp\left(i\phi \right)=\frac{1}{N}\overset{N}{\underset{j=1}{\sum }}\text{exp}(i\theta_{j}).$$ (7)

The amplitude of the centroid vector (indicated by the scalar r) represents the phase divergence or uniformity of N oscillators, and ϕ is the representative phase of the set of oscillators (Breakspear et al., 2010). Importantly, r describes the global phase coherence of the system at each time point as it disappears when the phases of oscillators have large circular variance and approaches 1 when all the oscillators are moving nearly in phase (Breakspear et al., 2010). It is customary to describe the global dynamic behavior of the ensemble using the mean and the standard deviation of r across time points, which are referred to as the global synchrony and global metastability, respectively (Cabral, Hugues, Sporns, & Deco, 2011; Váša et al., 2015). Metastability refers to the existence of a form of “winnerless competition” between two apparently opposing tendencies, namely, a tendency of individual oscillators to couple with each other and coordinate globally for multiple functions, and a tendency to be independent to express their specialized functions (Kelso, 2008; Roberts et al., 2018; Tognoli & Kelso, 2014). We computed global synchrony and global metastability as indicative of macroscopic coherence of the whole-brain network model, for all tested combinations of the model parameters. In addition, we calculated these measures for the empirical BOLD signals (Supplementary Figure 3). We calculated the distance between empirical and simulated global synchrony and metastability in order to evaluate their agreement.

### Perturbation Assessment

We simulated perturbation to every individual brain region in our model by shifting the dynamic regime of the targeted region from the oscillatory dynamics domain (characterized by the estimated bifurcation parameter) to the noise-driven fluctuations. With regard to investigating the effects from perturbations, we took advantage of the mathematical representation of the brain network as a graph with a set of nodes symbolizing brain regions and edges denoting the mutual interactions among nodes (Newman, 2003; Rubinov & Sporns, 2010; Strogatz, 2001).

#### Robustness—Network breakdown under random failures versus targeted attacks.

Robustness refers to the capacity of a system to absorb disturbances caused by either internal or external faults and still maintain its basic structure and function, even if some nodes and edges may be missing (Barabási, 2016). It has been shown that failure of hub regions in the brain (“targeted attacks”) have more detrimental effects on the network structure compared with “random failures” (Barabási, 2016). To test the performance of our model with regard to targeted attacks versus random failures, we simulated perturbation of every individual region by setting the bifurcation parameter to *a* = −2. An increasing fraction of regions, as denoted by *f*, were selected and perturbed, followed by measuring the size of largest strongly connected component (so-called giant component) formed in the network (Barabási, 2016). In an undirected graph, two nodes belong to the same component if there is at least one sequence of edges connecting them. The presence and absence of edges are denoted by binary edges. Therefore, giant component size was measured after applying a binary classification of the edges into two groups (0: disconnected or 1: connected) on the basis of a classification rule (i.e., threshold). We tried 100 different thresholds (0-1) for the binary classification of the static FC matrices, which were computed based on either the empirical BOLD data (empirical FC) or the simulated BOLD data (using optimal parameter set).

Next, we computed the accuracy of the binary classification test at every threshold (Supplementary Figure 4). Accuracy was determined by dividing the number of correct assessments (number of true positives + number of true negatives) into the number of all assessments (number of true/false positives + number of true/false negatives). Correct assessments refer to the functional connections which are classified as connected edges in both empirical and simulated FC matrices. As illustrated in Supplementary Figure 4, the application of conservative thresholds caused an increase in the accuracy of binarization at the expense of precision. Precision is the number of true positive assessments divided by the number of all positive assessments (number of true/false positives) returned by the classifier. Finally, after we fitted the cubic polynomial curve to the precision values (illustrated as the dotted green curve in Supplementary Figure 4), we estimated the location of the knee of both curves to be 0.27. Additionally, the intersection point of the two curves gives the threshold of 0.08, which is a more liberal threshold compared with the aforementioned knee point. It is worth mentioning that applying a common absolute threshold to the perturbation maps (as models of impaired brain connectome) seems to be preferred to relative thresholding (Fornito, Zalesky, & Bullmore, 2016).

Testing the robustness of our modeled brain network was performed using two different perturbation strategies: (a) Simulating random failures by applying perturbations to an increasing fraction of regions that were randomly selected. This procedure was repeated many times (*n* = 2,000) while the random number generator was reseeded at each iteration. (b) Simulating targeted attacks by applying perturbations to an increasing fraction of regions that were already sorted according to their degree of centrality in the structural connectivity matrix. That is, regions with a higher centrality were targeted for perturbation before the remaining regions. The degree of centrality of each region was measured as a composite hub score that was calculated by averaging the unity-based normalized measures of nodal strength, betweenness centrality, and closeness centrality (Freeman, 1977, 1978; Kaboodvand, Bäckman, Nyberg, & Salami, 2018; Rubinov & Sporns, 2010; Sporns, Honey, & Kötter, 2007; van den Heuvel & Sporns, 2013).

#### Vulnerability mapping—Assessment of the functional connectivity changes subject to distributed failures.

The susceptibility of a networked system to undergo significant changes in its function when confronted with different forms of disruption is called vulnerability. Applying perturbations to different regions of the network, and subsequently analyzing the perturbation patterns, is called vulnerability mapping, which aims to locate weaknesses within the system (Gollo et al., 2018). After applying the perturbation to every individual region of the brain network separately, we ended up with 68 sets of whole-brain network time courses, each set simulating the brain with malfunction in one of the 68 brain regions. In addition, we had one set of simulated signals corresponding to the healthy brain, which was modeled at optimal working point (see above). First, we computed the static FC matrices for every set of whole-brain signals (68 sets corresponding to simulated perturbations and one set for the optimal working point). Subsequently, we computed the nodal strength, followed by measuring the relative difference of nodal strength vector derived for every perturbation set, from the nodal strength vector yielded by the unperturbed set. Hence, we obtained 68 relative difference vectors, each representing the percentage change in nodal strength patterns caused by an induced single-node perturbation. Then, we aggregated the positive and negative values of percentage changes, separately. This was done in order to measure two different types of nodal vulnerability: nodal hyper-connectivity (bias to increase FC) and nodal hypo-connectivity (bias to decrease FC). We computed the average of positive and negative entities, representing measures of nodal hyper-connectivity and hypo-connectivity.

In addition, inter-network FC between each pair of functional networks (DM, LIM, CONT/dATT, SAL/vATT, SOM, and VIS networks) was computed by averaging FCs among all node pairs belonging to different functional networks (Kaboodvand et al., 2018). To compute the link vulnerability, we first subtracted the inter-network FC matrices derived for every perturbation set from the inter-network FC matrix yielded by the unperturbed set, followed by dividing the subtraction result into the FC matrix of the unperturbed set. Hence, we obtained 68 normalized divergence maps, each representing the percentage change in inter-network FC patterns caused by an induced single-node perturbation. Next, we aggregated the positive and negative values of normalized divergence maps, separately. This was done in order to measure two different types of link vulnerability: link hyper-connectivity and link hypo-connectivity risks. Therefore, we obtained measures of link hypo-/hyper-connectivity by averaging positive and negative entities independently.

#### Hazard mapping—Assessment of the hazardousness of different brain regions.

In addition to the three instantaneous phase-based measures that were used for finding the optimal model parameters, we calculated static FC-based measures including the global efficiency of whole-brain network, system-wise local efficiency, and the level of segregation for every functional system separately. Measures of the global and local efficiency were normalized by a matched (preserved degree distribution) random null model. Aforementioned measures were computed for the model of healthy brain (one set of whole-brain time courses corresponding to optimal simulated brain) as well as for 68 models of the impaired brain (68 sets of whole-brain time courses corresponding to simulated local perturbations). Each measure was unity normalized across 69 observations. Subsequently, static FC-based measures were recruited to create a 13-dimensional feature vector for every simulated perturbed/unperturbed set of whole-brain signals. The vector space associated with these 69 feature vectors is called the feature space. In a similar way, we created a 3-dimensional feature space for instantaneous phase-based measures.

If we apply a perturbation to any region of the brain, it is likely to cause a feature space divergence from the optimal simulated brain network. We refer to the relationship between the location of each perturbation and the level of distance in feature space as hazard mapping, while the measured distance indicates the degree of hazardousness for that particular location. We computed the pairwise Euclidean distance between the feature vectors obtained from the simulated perturbation sets and the feature vector of simulated healthy brain to investigate how every region’s failure contributes to increasing the dissimilarity between the simulated impaired brain and the simulated healthy brain. The level of distance was separately computed for the static FC-based measures and instantaneous phase-based measures. In the case of dynamic connectivity pattern, the Kolmogorov-Smirnoff distance was used as the numerical difference of their values. Afterwards, the two Euclidean distance measures were unity-based normalized and then summed together to create one distance measure for every perturbation.

A region shows a significant level of hazardousness if applying the perturbation to that particular region causes a substantial level of dissimilarity between the measures obtained from the simulated perturbation set and with the measures calculated for the simulated healthy brain. A brain region with the highest level of hazardousness may be interpreted as the region with the lowest level of fault tolerance.

## RESULTS

### Model Fitting and Frequency Dynamics

The parameters of the model (i.e., a, G, λ, and m) were estimated in a grid-search framework, extended to a four-dimensional space. We performed a grid search in two steps using a different granularity in each step. First, we used a coarse-grained search that spanned a wide range of values for each parameter. Then, we used the results from the first search to perform a second, more fine-grained search for the optimal choices of a, G, λ, and m. From the first search, we found evidence supporting our hypothesis that the resting brain operates at the edge of a critical point of bifurcation, with bifurcation parameters being close to 0 producing a better fit of the model. In the fine-grained grid search, frequency modulation coefficient m ranged from 0.1 to 0.2 in 11 steps, and frequency lethargy *λ* ranged from 0.2 to 1 with a step size of 0.2. The grid search for the global coupling G included 15 evenly distributed values in the interval of 0.002 to 0.03. The spanning range for the global bifurcation a included 71 values in the range from minus to plus 0.07.

**Figure 2.**
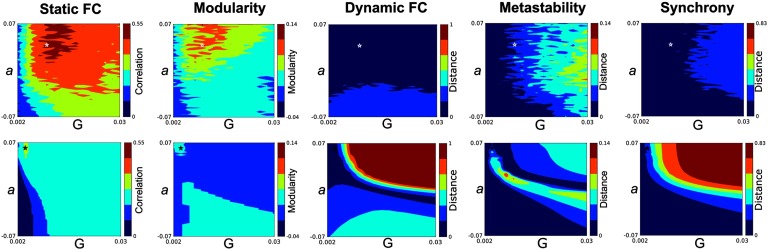
Parameter space search. This figure shows the exploration of the parameter space defined by the bifurcation parameter **a** and global coupling **G**, separately for our suggested frequency modulation-based model (first row) versus coupled Stuart-Landau oscillators (second row). Measures in the upper panel are reported at the optimal values of frequency lethargy λ = **0.4** and frequency modulation **m** = **0.4**. The first column depicts the Pearson correlation between empirical and simulated static FC patterns for different pairings of bifurcation parameter **a** and global coupling **G**. The second column shows the whole-brain modularity computed for the simulated static FC matrix. The Kolmogorov-Smirnoff distance between the similarities of coherence measures, obtained from the empirical BOLD data and simulated signals, as well as the differences of metastability and synchronization of simulated signals from the average metastability and synchronization measures of empirical data, are respectively illustrated in columns 3–5. Measures associated with the optimal choice of global coupling (**G**), bifurcation parameter (**a**), frequency lethargy **λ**, and frequency modulation **m** are shown as a white asterisk in the upper panel (**G** = **0.01**, **a** = **0.038**, **λ** = **0.4** and **m** = **0.14**), whereas the optimal point for the classic coupled Stuart-Landau oscillators is depicted by a black asterisk in the lower panel (**G** = **0.004**, **a** = **0.062**). See Supplementary Figure 5 for corresponding parameter space search including frequency lethargy **λ** and frequency modulation **m**.

Figure 2 illustrates the grid-search landscapes of global bifurcation a and global coupling G for five different measures of interest, separately for our proposed frequency modulation-based model versus coupled Stuart-Landau oscillators. The optimal choice of global coupling (G), bifurcation parameter (a), frequency lethargy *λ*, and frequency modulation m is shown as a white asterisk in the first panel of Figure 2 (G = 0.01, a = 0.038, λ = 0.4, and m = 0.14). The optimal parameter set is where the level of modularity of the model and the Pearson correlation between static FC derived from the model and empirical data is high. At the same time, the Kolmogorov-Smirnoff distance between the similarities of coherence measures, obtained from empirical BOLD data and simulated signals, as well as the differences of metastability and synchronization of simulated signals from the average metastability and synchronization measures of empirical data are considerably low. In order to provide evidence that an increased number of model parameters is justified by goodness of fit, we computed the Akaike information criterion (AIC). Using the optimal parameter set, we recomputed the aforementioned composite similarity score separately for each individual’s empirical BOLD signals as a reference. The resultant distribution of composite scores was used to estimate the maximum value of the likelihood function for the model. Our results showed that the frequency modulation-based model had smaller absolute AIC value (−637.41) and performs better than the coupled Stuart-Landau oscillators (−889.28).

**Figure 3.**
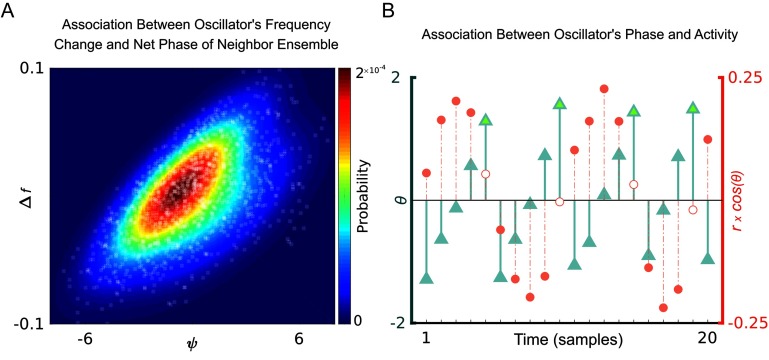
Frequency dynamics in the proposed model of brain oscillations. (A) There is a positive association between the frequency change of an oscillator and net phase of its neighbor ensemble. The oscillator modeling the right precuneus cortex was selected for illustration purpose. The probability distribution for the Gaussian mixture model across two components (i.e., net phase of neighbor ensemble and change of frequency) is depicted in the first panel. (B) Association between the phase (*θ*) of the oscillator modeling the left posterior cingulate cortex (an example neighbor of the right precuneus) and its level of activity (r × cos *θ*). The phase of oscillator begins to grow when the magnitude of its activity starts to shrink and it approaches the maximum value (illustrated by light green upward-pointing triangles in Figure 3B) when the magnitude of its activity is going to pass the 0 value (illustrated by empty circles in Figure 3B).

In the proposed model of the brain oscillations, the frequency of each oscillator is modulated by the net phase of the neighbor ensemble. Figure 3A shows the positive association between net phase of the neighbor ensemble and change of frequency in the case of right precuneus (chosen for illustration purposes). The frequency of an oscillator undergoes the highest change when the net phase of the neighbor ensemble reaches its extremum. In other words, the highest frequency change rate is yielded for oscillator *j* when the net phase of its neighbor ensemble ψ_j_ = ∑ C_ij_ *θ*_*i*_ reaches its extremums. On the other hand, the phase of each neighbor oscillator (denoted by *θ*_*i*_) is related to the activity of that particular oscillator (i.e., *r*_*i*_ cos *θ*_*i*_). Figure 3B illustrates how the phase of the left posterior cingulate cortex (an example of a neighbor of the right precuneus cortex) is associated with its activity. The phase of oscillator begins to grow when the magnitude (i.e., absolute value) of its activity starts to shrink, and it approaches the maximum value (illustrated by light green upward-pointing triangles in Figure 3B) when the magnitude of its activity is passing 0 (illustrated by empty circles in Figure 3B). In other words, when the absolute value of overall activity in the neighborhood of oscillator *j* starts to decline, the net phase of the neighbor ensemble (ψ_j_) begins to grow until the overall neighborhood activity passes 0 and starts to regrow again. Therefore, the net phase of the neighbor ensemble (ψ_j_) reaches its maximum value at the point where the overall neighborhood activity is at the minimum level. In conclusion, the frequency of an oscillator undergoes the highest change when the net activity of its neighbor ensemble is in the vicinity of 0, so that the speed of frequency changes reaches its maximum positive value (speedup) when the net neighborhood activity gets close to passing the zero line, whereas it gets its maximum negative value (slowdown) when the net neighborhood activity has just started to grow.

### Robustness, Vulnerability, and Hazard Mapping for the Whole-Brain Network Model

**Figure 4.**
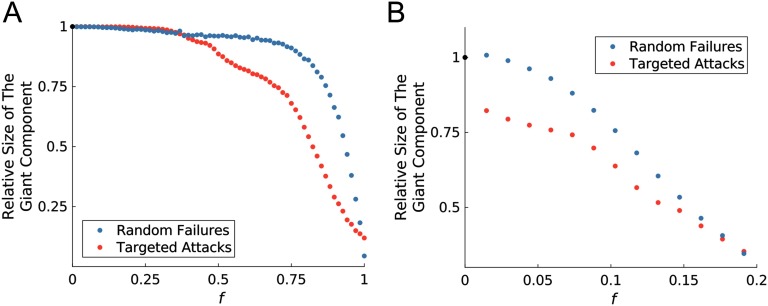
Network breakdown under random failures versus targeted attacks is illustrated for both a liberal (absolute threshold = 0.08) and restrictive (absolute threshold = 0.27) thresholding strategies, respectively in panels A and B. In the case of random failures, the fraction of nodes that belong to the giant component is computed after an *f* fraction of nodes are randomly selected for perturbation. For a targeted attack, we calculate the fraction of nodes that belong to the giant component after an *f* fraction of nodes are perturbed in a decreasing order of their hub score, so that we start with the node with highest hub score, followed by the next highest, and so on. The procedure of simulating random failures was repeated 2,000 times, which resulted in a smoothed plot. However, the Savitzky-Golay filter was applied to the values from targeted attacks, to enable the reader to easily see the general pattern of decline.

As a measure of network robustness, Figure 4 displays the fraction of brain regions that belong to the giant component after applying either random or targeted perturbations to an *f* fraction of regions. The size of the giant component at every value of *f* was divided by the actual size of the giant component, which provides a relative measure of giant component size. From Figure 4 it can be seen that in the face of random failure, the fragmentation process is gradual. However, the whole-brain network has a lower tolerance when faced with selective attacks to hub regions. Thus, our simulated brain network shows a lower degree of robustness for targeted attacks versus random failures.

**Figure 5.**
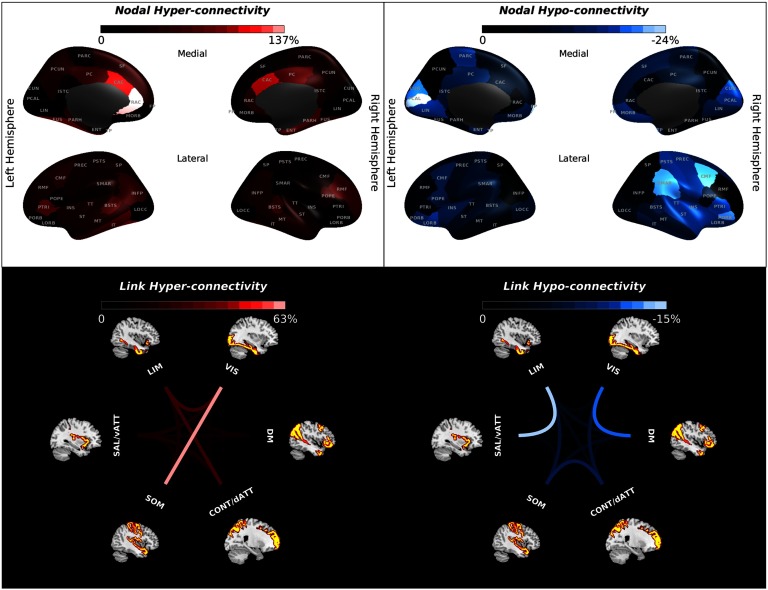
Vulnerability mapping. The upper panel illustrates the vulnerability of different regions. The level of hyper-connectivity risk, which measures the tendency to increase FC in face of distributed failures in the whole-brain network, is depicted in the left column, whereas the hypo-connectivity rate as indicative of the tendency to have decreased FC in the face of distributed failures in the whole-brain network is shown in the right column. The upper panel of this figure shows the nodal risk rate for hyper- and hypo-connectivity in color format for every node listed in Supplementary Table 1. The lower panel of this figure illustrates the vulnerability of inter-network FCs by color-coded links between different resting-state networks. See Supplementary Table 1 for a list of the abbreviated node names. DM, default mode; LIM, limbic; dATT/CONT, dorsal attention or control; SAL/vATT, salience or ventral attention; SOM, somatomotor; VIS, visual.

Applying an in silico perturbation protocol to our proposed model enabled us to quantify network vulnerability in the form of hypo-/hyper-connectivity risk rate for either different brain regions (Figure 5; upper panel) or FCs between different functional systems (Figure 5; lower panel). Subregions of the cingulate cortex, the temporo-parietal junction (i.e., banks superior temporal sulcus), the parahippocampal cortex (encompassing the parahippocampal gyrus and the fusiform gyrus), the inferior temporal gyrus, the middle frontal gyrus, and the inferior parietal cortex (including the inferior parietal gyrus and the angular gyrus) showed a strong risk for hyper-connectivity (Figure 5; upper panel, left column). Also, the posterior subdivision of inferior frontal gyrus (pars opercularis) in the right hemisphere as well as the middle subdivision of inferior frontal gyrus (pars triangularis) in the left hemisphere had a strong tendency to increase FC (Figure 5; upper panel, left column). On the other hand, we observed that the regions with the strongest hypo-connectivity risk were located in the posteromedial visual system, frontal pole, medial and lateral orbital frontal cortex, right supramarginal gyrus, right inferior frontal gyrus (pars triangularis and pars orbitalis), and right caudal middle frontal gyrus, and to a lesser extent in the right sensory-motor cortex (postcentral and precentral gyrus), the right insular cortex, and the right superior temporal gyrus as well as left paracentral lobule (Figure 5; upper panel, right column). Furthermore, we observed the highest hyper-connectivity risk for the FC of the VIS system with the SOM and LIM systems, as well as FC of the CONT/dATT system with the LIM system (Figure 5; lower panel, left column). Additionally, we observed considerable hyper-connectivity risk for the FC between attentional subsystems (SAL/vATT and CONT/dATT), as well as between the SAL/vATT system and the VIS and DM systems, and to a lesser extent between the LIM and SOM systems. The strongest hypo-connectivity risks were found for the FC between the DM and VIS systems, and the LIM and SAL/vATT systems, and between the CONT/dATT system with SOM and VIS systems (Figure 5; lower panel, right column).

**Figure 6.**
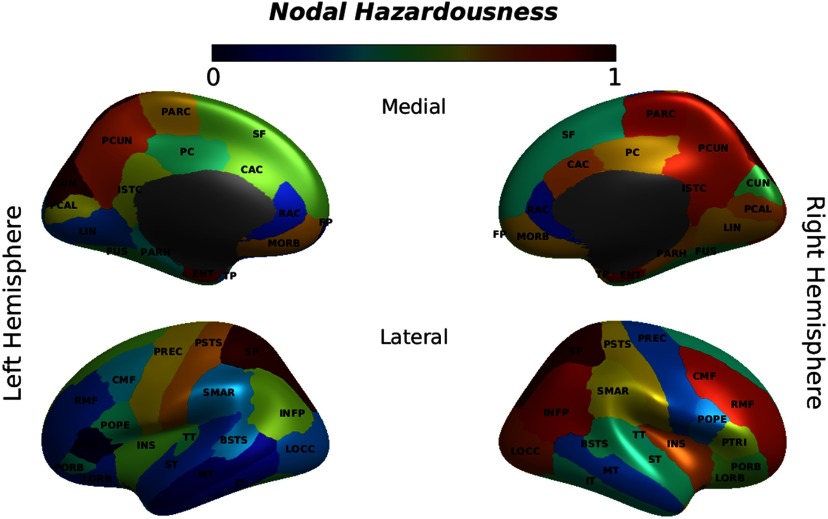
Hazardousness mapping. The degree of hazardousness for a region/node was measured as the degree of feature space divergence from the optimal simulated brain network caused by applying perturbation to that particular node. Normalized measures of distance in feature space are depicted in color format for every node as listed in Supplementary Table 1. See Supplementary Table 1 for a list of the abbreviated node names.

Finally, we examined the effects of malfunctions in different brain regions when a combination of local and global measures of brain network were taken into account (hazardousness mapping). The results of the hazardousness mapping are shown in Figure 6. In relating the hazardousness measures and the key nodal centrality measures, we found a significant correlation for the clustering coefficient (r = −0.33, *p* = 0.008), degree (r = 0.355, *p* = 0.006), and strength (r = 0.39, *p* = 0.004). Measure of association with hazardousness for the local efficiency was not significant (r = −0.20, *p* = 0.094). Reported *p* values are FDR-corrected across four comparisons.

The analysis of the size of the damage inflicted on the network by applying perturbation to individual nodes showed that the superior parietal cortex (also known as the dorsal attention system) and the left cuneus cortex, and to a lesser extent the precuneus cortex and the entorhinal cortex, were the most critical regions for maintaining adequate network communication. In addition, we observed a high level of hazardousness for some regions in the right hemisphere such as the inferior parietal cortex (including the inferior parietal gyrus and the angular gyrus), the isthmus cingulate cortex, the middle frontal gyrus, the insular cortex, and the caudal anterior cingulate cortex. Upon closer inspection, the medial orbital frontal cortex and the right posterior cingulate cortex, as well as the left postcentral gyrus and right parahippocampal gyrus, had some level of hazardousness, too (Figure 6). Malfunction in any of the aforementioned areas resulted in a considerable divergence in the brain network characteristics.

## DISCUSSION

### Frequency Dynamics

Based on our constructed model, the oscillatory frequencies undergo the highest change when the activity in the neighborhood is in the vicinity of 0 (Figure 3B). In other words, the slope of frequency change takes its maximum positive value (i.e., speeds up) when the input signals to that region are subsiding, while it experiences a slowdown when the input signals start to grow. Intuitively, this phenomenon may be likened to tuning a radio receiver, to enable it to receive incoming signals from the neighborhood. Supplementary Figure 6b illustrates the working frequency range of oscillators.

Of note, we know from the literature that the frequency spectrum of intrinsic oscillations of the brain has a time-varying nature (C. Chang & Glover, 2010; Yaesoubi et al., 2015), and notably we had hypothesized that the regional frequency is modulated by the activity of its neighbor regions. We were inspired by two famous electrophysiology theories; first, neurons acting as integrators, so that they sum or average their inputs to generate action potentials (Abeles, 1982; König, Engel, & Singer, 1996; Salinas & Sejnowski, 2001); second, neurons being exquisitely sensitive to certain temporal input patterns, giving rise to oscillatory activity or switching from one oscillatory regime to another (Salinas & Sejnowski, 2001). Moreover, a previous science paper (Mukamel et al., 2005) has revealed that fMRI signals can provide a reliable measure of the firing rate of human cortical neurons.

Our model simulates ultraslow oscillations. The power spectral density as well as the distribution of estimated intrinsic frequencies of these oscillations have been presented in Supplementary Figure 6a. Of note, the objective of our modeling study was mainly to reproduce the interrelationships (i.e., FC and instantaneous coherence) of the human brain, not the frequency dynamics of the individual oscillators, notably only based on a fixed structural connectome (without modifying SC using the FC). To tackle this, we used a cost function that was based on interregional associations in the empirical data and not the intrinsic frequency, given a fixed SC matrix and a fixed level of activity for all brain regions (i.e., one common bifurcation parameter). Otherwise, tuning the local bifurcation parameters to reproduce the region-specific proportions of power in a narrow band (0.04–0.07 Hz; for example, see Senden et al., 2017) could increase the similarity between frequency characteristics of the simulated data and the empirical data, at the cost of introducing the possibility of overfitting due to optimization of several local bifurcation parameters for different brain regions. Furthermore, using one common bifurcation parameter for all the different oscillators seemed the best fit for our later aim of applying in silico perturbations to the model.

### Perturbation Assessment

We simulated the effects of brain lesions by shifting the associated regions’ dynamical regime to elicit noisy behavior, rather than removing nodes or links. A comparable protocol was previously used to temporarily either promote or disrupt synchronization in random brain regions and subsequently assess the recovery back to baseline (Deco et al., 2018). Of note, a previous study that optimized the region-specific (also known as local) bifurcation parameters by fitting the normalized power for each intrinsic frequency band to the normalized power observed for empirical signal could provide evidence that most brain regions in patients with Parkinson’s disease had negative values of the bifurcation parameter, representing neuronal noise corresponding to an asynchronous firing of neurons (Saenger et al., 2017).

#### Robustness of the whole-brain network model.

We observed that our model of the brain has a lower tolerance when facing selective attacks to central regions. However, in the face of random failures, the fragmentation process was gradual. To the best of our knowledge, no previous study has evaluated robustness of the brain (here measured as giant component size) by perturbing regions in silico. Removing nodes or links (as a way to simulate effects of structural lesions) is the most commonly used method for investigation of brain robustness. Since hub scores were calculated based on the actual structural connectome and perturbations were simulated by applying bifurcation-induced shifts in the dynamical regime, this finding may further validate the model and the recruited perturbation strategy presented here.

#### Hazardousness mapping.

The hazard rates induced by primary failures of individual nodes provide relevant insights not only into the size of the damage inflicted on the network by individual nodes, but also into the potential origins of disease. Our results regarding nodal hazardousness rates are interesting in the context of the question of why failures in some cases might lead to long-lasting impairment and disease, while others might not. To this end, we observed the highest level of hazardousness for the CONT/dATT system, which mainly covers the superior parietal cortex, the right caudal middle frontal gyrus, and the bilateral rostral middle frontal gyrus, as well as for the posteromedial subsystem of the DM network (particularly the precuneus cortex, the inferior parietal cortex, and the isthmus cingulate cortex). In addition, the LIM system, in particular the entorhinal cortex, was found to be a hazardous region. The SAL/vATT system (mostly including the medial subdivisions like posterior cingulate cortex and caudal anterior cingulate, but also the insular cortex) was also found to cause substantial levels of damage in case of malfunction (Figure 6). These results substantiate the important role of aforementioned regions for global coordination of information flow across the whole-brain network.

It is also of interest to relate our observation of a high level of hazardousness for the posteromedial subsystem of the DM network to its important role in integrating the bottom-up attention with behaviorally related information from memory and perception (Andrews-Hanna, Smallwood, & Spreng, 2014). Indeed, previous studies have suggested that the ventral posteromedial cingulate cortex (referred to as the isthmus cingulate cortex in the Desikan-Killiany atlas) has dense FC that essentially is restricted to the DM network (Kaboodvand et al., 2018; Leech, Kamourieh, Beckmann, & Sharp, 2011; Leech & Sharp, 2014; Spreng, Sepulcre, Turner, Stevens, & Schacter, 2013; Tomasi & Volkow, 2010). Hence, it may be said that it serves as the key provincial hub for the DM network, in that it mediates a large proportion of the traffic that facilitates interactions within this system (Kaboodvand et al., 2018). Moreover, the precuneus/posterior cingulate cortex has been identified as a critical connector hub (Achard, Salvador, Whitcher, Suckling, & Bullmore, 2006; Fransson & Marrelec, 2008; Leech et al., 2011; Spreng et al., 2013; van den Heuvel & Sporns, 2013; Zuo et al., 2012), with dense connections spreading across the whole-brain network (Fransson & Marrelec, 2008; Hagmann et al., 2008). FC patterns of the posteromedial subsystem of the DM network have a well-established association with mild cognitive impairment and Alzheimer’s disease (Bai et al., 2009; Buckner et al., 2005; Celone et al., 2006; Greicius, Srivastava, Reiss, & Menon, 2004). Positron emission tomography imaging in Alzheimer’s disease has shown high amyloid-*β* deposition in the prominent hubs located in posteromedial cingulate, lateral temporal, lateral parietal, and medial/lateral prefrontal cortices (Buckner et al., 2009; Hedden et al., 2009). There is some evidence suggesting that high level of connectedness that is required for large information transfer in the hubs may cause in augmentation of the underlying pathological cascade in AD (Buckner et al., 2009).

Given that our finding of regions with high hazardousness in the parietal association cortex was predominately right lateralized, it is worthwhile to inquire about the reason for this asymmetry. Indeed, previous literature suggests that lesions of the parietal association cortex in the right hemisphere are more detrimental, whereas that left parietal lesions tend to be compensated by the intact right hemisphere (Purves et al., 2004). Notably, contralateral neglect syndrome is specially associated with the damage to the right parietal association cortex. There is evidence suggesting that the right parietal cortex mediates attention to both left and right halves of the body and the extrapersonal space, whereas the left hemisphere mediates attention primarily to the right side (Purves et al., 2004). There is also strong evidence for the belief that the right middle fontal gyrus is a site of convergence of the dorsal and ventral attention systems, by acting as a circuit breaker for interrupting ongoing endogenous attentional processes in the dorsal network and reorienting a person’s attention to an exogenous task-relevant stimulus (Corbetta, Kincade, & Shulman, 2002; Fox, Corbetta, Snyder, Vincent, & Raichle, 2006; Japee, Holiday, Satyshur, Mukai, & Ungerleider, 2015). Therefore, the right middle fontal gyrus has control over both ventral attention and dorsal attention networks and would be responsible for the flexible modulation of internal and external attention (Corbetta et al., 2002; Fox et al., 2006; Japee et al., 2015).

We found a high level of hazardousness for the entorhinal cortex. This finding resonates well with the previous studies that have shown that impairment of the entorhinal cortex is persistently reported in patients with Alzheimer’s disease (Van Hoesen, Hyman, & Damasio, 1991), schizophrenia (Arnold, Ruscheinsky, & Han, 1997), as well as in cases of traumatic brain injury and stroke. Moreover, entorhinal damage is believed to cause interference with sensory integration and also cause memory deficits (in particular spatial learning impairment; Van Hoesen et al., 1991). Moreover, it has been suggested that the age-related entorhinal cortex thinning occurs before hippocampal volume loss and FC decline in the DM system, which impacts communication between medial temporal lobe with the cortical DM system, contributing to age-related memory deficits (Tisserand, Visser, van Boxtel, & Jolles, 2000; Ward et al., 2015). Of note, it was recently discussed that the parahippocampus and retrosplenial cortex (referred as the isthmus cingulate cortex in the Desikan-Killiany atlas) are sequential interfaces between the medial temporal lobe subsystem of the DM system and cortical regions of the DM system (Kaboodvand et al., 2018). In particular, it was shown that the entorhinal cortex mediates the interaction between the medial temporal lobe and the retrosplenial cortex. Therefore, the dynamic coupling/decoupling of the medial temporal lobe from the cortical DM system (Huijbers, Pennartz, Cabeza, & Daselaar, 2011; Vannini et al., 2011; Young & McNaughton, 2009) is mediated by the parahippocampus and the retrosplenial cortex together (Kaboodvand et al., 2018). Thus, previous research on the important role for the parahippocampus, including entorhinal cortex in the brain’s network is in agreement with our findings of a high level of hazardousness for this region.

Additionally, we found a considerable level of hazardousness for right anterior cingulate cortex. The anterior cingulate cortex is one of the key integrative brain hubs (Lavin et al., 2013), and it has been suggested to play important role in high-level cognitive processing, outcome monitoring, action planning, and emotion processing (Fernández-Matarrubia et al., 2018; Lavin et al., 2013). Furthermore, it acts as a core component in fronto-striatal circuitry. There is evidence that abnormal functioning of right anterior cingulate may have a pathophysiologic role in speech impairment (C.-C. Chang, Lee, Lui, & Lai, 2007) and in the cognitive and emotional impairment related to attention deficit hyperactivity disorder (Tian et al., 2006), schizophrenia (H. Yan et al., 2012), and panic disorder (Shinoura et al., 2011). Notably, there is some evidence in support of FC changes between the medial frontal and anterior cingulate in different dementia groups (Fernández-Matarrubia et al., 2018).

#### Vulnerability mapping.

In response to distributed malfunctions (i.e., heterogeneous locations of injury), we observed that functional connections of our brain are obviously more susceptible to hyper-connectivity than hypo-connectivity, particularly in brain regions with high connectedness. Previous studies have repeatedly reported increased functional connectivity after traumatic brain injury as a compensatory brain response to make up for physiological disturbances (Bharath et al., 2015; Hillary et al., 2014; Iraji et al., 2016; Nakamura, Hillary, & Biswal, 2009), particularly so for regions with high connectedness (Hillary et al., 2014). Basically, the observation that “the rich get richer” is in line with the preferential attachment theory underlying scale-free network development, which suggests that new connections mostly occur in the regions with high connectedness (Barabási, 2016). Accumulating evidence has suggested that hyper-connectivity is a common response to neurological disruption in brain insults such as traumatic brain injury, mild cognitive impairment, Alzheimer’s disease, multiple sclerosis, and epilepsy (Hillary et al., 2014, 2015). Our nodal vulnerability analysis revealed that the most prominent cases for hyper-connectivity belong to the regions in the default mode, salience, and control systems; more specifically, the anterior cingulate cortex, the inferior parietal cortex, the left precuneus cortex, and also the posterior cingulate cortex and the middle frontal gyrus, which is consistent with the previous literature on traumatic brain injury (Hillary et al., 2014; Iraji et al., 2016; Mayer, Mannell, Ling, Gasparovic, & Yeo, 2011). For example, increased connections in the acute phase of injury have been reported for the left cingulate gyrus, the left precuneus, and the right prefrontal cortices (Bharath et al., 2015; Hillary et al., 2014).

Our results of high vulnerability in the frontoparietal regions is perhaps not overly surprising given that frontoparietal regions are involved in the top-down attentional control. Interestingly, hyper-connectivity of frontoparietal regions in traumatic brain injury patients is attributed to the increased awareness of the external environment. These observations may explain the reports of cognitive fatigue in these patients (Bharath et al., 2015; Muller & Virji-Babul, 2018; Shumskaya, Andriessen, Norris, & Vos, 2012).

Moreover, previous results suggest that the traumatic brain injury–related hyper-connectivity of the DM system is beneficial for cognitive function (Sharp et al., 2011), in that patients with stronger FC showed the least amount of cognitive impairment. A previous longitudinal study found that during the recovery phase after injury, the weight of network connections diminishes. This finding implies that over the course of recovery, the functional connectome of the injured brain begins to approximate the healthy functional connectome (Nakamura et al., 2009).

Furthermore, there are reports of the decreased FC in the literature that are predominantly right-sided, involving the right frontal and parietal regions (Bharath et al., 2015; Borich, Babul, Yuan, Boyd, & Virji-Babul, 2015; Mayer et al., 2011). For example, the hypo-connectivity for the right supramarginal gyrus and the right middle frontal gyrus has been reported for patients with mild brain injury (Borich et al., 2015; Mayer et al., 2011). The lack of dynamic flexibility after traumatic brain injury has been linked to the observed hypo-connectivity of right frontal eye field (Muller & Virji-Babul, 2018).

Our vulnerability mapping suggests that the SAL/vATT system, the CONT/dATT system, and the DM system included the regions that had the highest hyper-connectivity risks (e.g., the posterior/anterior cingulate cortices, the rostral middle frontal, and the inferior parietal cortex). On the other hand, we found that the medial visual network, regions of the right parietal lobe (the right supramarginal gyrus and postcentral gyrus), as well as regions of the frontal lobe (caudal middle frontal gyrus, medial and lateral orbital frontal cortex, frontal pole, pars orbitalis subdivision of the inferior frontal gyrus) had noticeable risks of hypo-connectivity.

Our results regarding the vulnerability of intersystem connections are also in line with the published literature on the effects of injury to brain networks. For example, a previous study of mild traumatic brain injury has reported functional hyper-connectivity for the FC of posterior cingulate cortex with the frontal eye fields, the dorsolateral prefrontal cortex, the associative visual cortex, the somatosensory association cortex, and the premotor cortex, as well as for the FC of occipital lobe with the frontal lobe (Iraji et al., 2016). Our results also indicated a considerable level of hyper-connectivity for the SAL/vATT system, encompassing the posterior cingulate cortex. Of note, there is a body of evidence regarding the hyper-connectivity within the frontal lobe (Bharath et al., 2015; Borich et al., 2015; Hillary et al., 2014; Mayer et al., 2011).

These previous clinical observations give some support to our results of significant hyper-connectivity risks for the FC of SAL/vATT system (which encompasses the posterior cingulate cortex) with the DM, CONT/dATT, SOM, and VIS systems, as well as strong hyper-connectivity between the VIS and LIM systems. Moreover, previous reports of the hyper-connectivity within the frontal lobe (Bharath et al., 2015; Hillary et al., 2014) are of relevance to our observation of strong hyper-connectivity between the CONT system and the limbic or the SAL/vATT systems. Abnormal hyper-connectivity between the top-down attentional systems has been regarded as the underlying cause for some posttraumatic brain injury symptoms, such increased distractibility and cognitive fatigue (Borich et al., 2015; Mayer et al., 2011; Shumskaya et al., 2012).

Taken together, our findings regarding hyper-/hypo-connectivity risks in response to distributed brain failures are largely in agreement with the observations reported in the aforementioned clinical studies. Thus there are good reasons to believe that the in silico perturbation assessment of the whole-brain dynamical connectome can provide valuable information in predicting reorganization of brain connectivity in response to neurological dysfunctions. In addition, we provided novel evidence for dissimilar susceptibility of FCs when facing heterogeneous failures in the brain network. Moreover, our proposed hazardousness map may serve as a useful index for predicting the impact of specific failures on network characteristics and a particular damage’s contribution in potential long-lasting impairment and disease (Barabási, 2016).

## ACKNOWLEDGMENTS

Data were provided by the Human Connectome Project, WU-Minn Consortium (principal investigators: David Van Essen and Kamil Ugurbil; 1U54MH091657), funded by the 16 NIH institutes and centers that support the NIH Blueprint for Neuroscience Research; and by the McDonnell Center for Systems Neuroscience at Washington University. The funders had no role in study design, data collection and analysis, decision to publish, or preparation of the manuscript. The authors thank Behzad Iravani for his valuable methodological advice.

## SUPPORTING INFORMATION

Supporting information for this article is available at https://doi.org/10.1162/netn_a_00104.

## AUTHOR CONTRIBUTIONS

Neda Kaboodvand: Conceptualization; Data curation; Formal analysis; Investigation; Methodology; Project administration; Resources; Software; Validation; Visualization; Writing – Original Draft; Resources; Writing – Review & Editing. Martijn P. van den Heuvel: Data curation; Formal analysis; Resources; Writing – Review & Editing. Peter Fransson: Conceptualization; Funding acquisition; Project administration; Resources; Supervision; Validation; Writing-Orginal Draft; Writing – Review & Editing.

## FUNDING INFORMATION

Peter Fransson, Swedish Research Council (http://dx.doi.org/10.13039/501100004359), Award ID: 2016-03352. Peter Fransson: Swedish e-Science Research Center. Martijn P. van den Heuvel, Mental Health and Quality of Life (MQ). Martijn P. van den Heuvel, Netherlands Organisation for Scientific Research (NWO), VIDI grant (452-16-015). Martijn P. van den Heuvel, Netherlands Organisation for Scientific Research (NWO), ALW open grant (ALWO179).

## TECHNICAL TERMS

Robustness: The capacity of a system to absorb disturbances and still maintain its basic structure and function.
Time-varying (also referred to as dynamic) functional connectivity: A moment-to-moment measure of functional connectivity, which captures fluctuations in the interactions over time.
Vulnerability: The susceptibility of a network component to undergo significant change in its function when confronted with any malfunction in the network.
Hazardousness: The degree of functional network disturbance caused by malfunction in a particular network component.
Hopf bifurcation: A phenomenon that occurs as the stability of a fixed point switches and a periodic solution appears.
Bifurcation: An abrupt qualitative change in the behavior of a system caused by applying a slight change to a system parameter.
Nonlinear dynamical systems: Dynamic systems governed by a set of equations in which temporal changes in variables cannot be written as a linear combination of the system variables.
Fixed point or equilibrium: Any point where there is no flow or change in the state variable of the dynamic system (time-independent solution).
Trajectory: A solution of the differential equation, given an arbitrary initial condition, that represents the time evolution of the system in the phase-space.
Phase-space: A collection of all possible states of the system, where each state refers to a set of system variables that best characterizes the system.
Model fitting: Estimating optimal model parameters by maximizing the similarity between the empirical and simulated data.
