# Supplementary material for: Adaptive frequency-based modeling of whole-brain oscillations: Predicting regional vulnerability and hazardousness rates
Source: Netw Neurosci. 2019 Sep 1;3(4):1094–120. doi: 10.1162/netn_a_00104 (PMC6779267; doi:10.1162/netn_a_00104)
Supplement: Supplementary file 1 [file netn-03-1094-s001.pdf]

Kaboodvand, N., van den Heuvel, M. P., & Fransson, P. (2019). Supporting information for "Adaptive frequency-based modeling of whole-brain oscillations: Predicting regional vulnerability and hazardousness rates." *Network Neuroscience*, *3*(4), 1094–1120. https://doi.org/10.1162/netn_a_00104

Supplementary Material

Supplementary Equations. *The optimal working point of the model was estimated using a composite distance score which was calculated as the average of five unity-based normalized distance measures, as below:*

$$Composite\ Distance\ Score = \left(\left|UN\left(synchrony_{Sim} - \overline{synchrony_{emp}}\right)\right| + \left|UN\left(metastability_{Sim} - \overline{metastability_{emp}}\right)\right| + |UN(\mathrm{KSD})| + |1 - UN(\mathrm{Q})| + |1 - UN(\mathrm{CORR})|\right)/5$$

$$UN\ (x) = \frac{x - \min\ (x)}{\max(x) - \min\ (x)}$$

* UN, Unity normalization; KSD, Kolmogorov-Smirnoff distance between the similarities of coherence measures, obtained from the empirical BOLD data and simulated signals; $CORR$, Pearson correlation coefficient between the empirical FC matrix and the FC matrix obtained for the simulated signals; Q, Whole brain modularity.

Supplementary Table 1. *The list of abbreviations and complete names for all 68 regions-of-interest as included in the 'Desikan-Killiany' cortical atlas are shown in the table.*

Abbreviation	Complete Name
**BSTS**	Banks superior temporal sulcus
**CAC**	Caudal anterior-cingulate cortex
**CMF**	Caudal middle frontal gyrus
**CUN**	Cuneus cortex
**ENT**	Entorhinal cortex
**FUS**	Fusiform gyrus
**INFP**	Inferior parietal cortex
**IT**	Inferior temporal gyrus
**ISTC**	Isthmus – cingulate cortex
**LOCC**	Lateral occipital cortex
**LORB**	Lateral orbital frontal cortex
**LIN**	Lingual gyrus
**MORB**	Medial orbital frontal cortex
**MT**	Middle temporal gyrus
**PARH**	Parahippocampal gyrus
**PARC**	Paracentral lobule
**POPE**	Pars opercularis
**PORB**	Pars orbitalis
**PTRI**	Pars triangularis
**PCAL**	Pericalcarine cortex
**PSTS**	Postcentral gyrus
**PC**	Posterior-cingulate cortex
**PREC**	Precentral gyrus
**PCUN**	Precuneus cortex
**RAC**	Rostral anterior cingulate cortex
**RMF**	Rostral middle frontal gyrus
**SF**	Superior frontal gyrus
**SP**	Superior parietal cortex
**ST**	Superior temporal gyrus
**SMAR**	Supramarginal gyrus
**FP**	Frontal pole
**TP**	Temporal pole
**TT**	Transverse temporal cortex
**INS**	Insular cortex

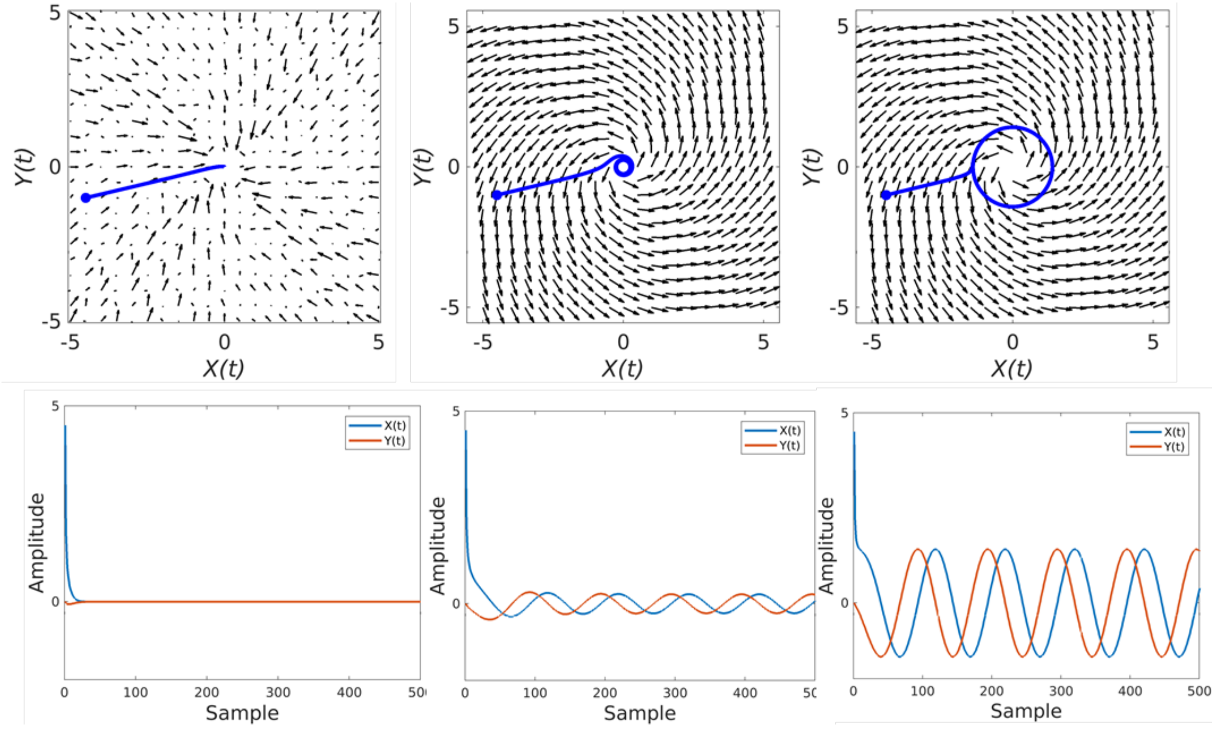

Supplementary Figure 1. *An illustrative example of phase space vector fields as well as temporal evolution of the Stuart-Landau oscillator. where* $\boldsymbol{z} = \boldsymbol{r}\, \boldsymbol{e}^{\boldsymbol{i\theta}} = \boldsymbol{x} + \boldsymbol{iy}$ *is a complex number describing the state of the oscillator,* $\boldsymbol{\omega} \in \mathbb{R}$ *is the frequency of each oscillator and the bifurcation parameter* $\boldsymbol{a} \in \mathbb{R}$ *determines whether the oscillator is characterized by noisy fluctuations or exhibits oscillatory behavior. The origin (i.e.,* $\mathbf{z} = \mathbf{0}$*) is the fixed point of this system. The eigenvalues of this system all have complex conjugate values (*$\boldsymbol{\sigma} = \boldsymbol{a} \pm \boldsymbol{i\omega}$*), indicating a spiral trajectory behavior in the neighborhood of the fixed point in phase space. If* $\mathbf{a} < \mathbf{0}$*, then the origin is a stable equilibrium solution with solutions spiraling into the origin (as illustrated in the first column of the figure). However, if* $\mathbf{a} > \mathbf{0}$*, then the origin is an unstable equilibrium with solutions spiraling out from the origin (as illustrated in the third column of the figure). The illustrated closed orbit in the phase space represents the periodic behavior of the system. Solutions that reside inside of the closed orbit will spiral out towards the orbit, while solutions outside of the orbit will spiral inward. The middle column includes an example phase space and the associated signals at the bifurcation point (*$\mathbf{a} = \mathbf{0}$*).*

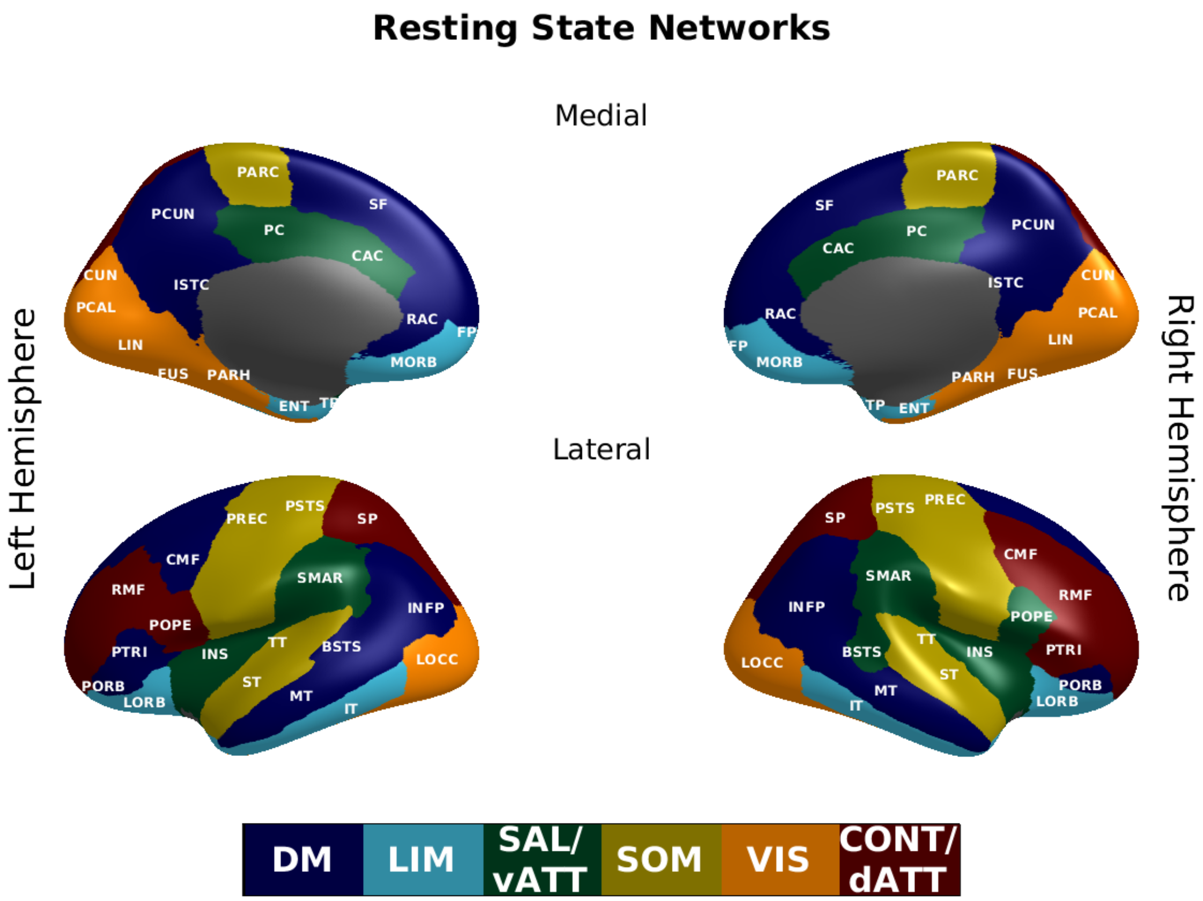

Supplementary Figure 2. *The layout of the functional networks. The used abbreviations for brain regions are shown in Supplementary Table 1. DM, default mode; LIM, limbic; dATT/CONT, dorsal attention or control; SAL/vATT, salience or ventral attention; SOM, somatomotor; VIS, visual.*

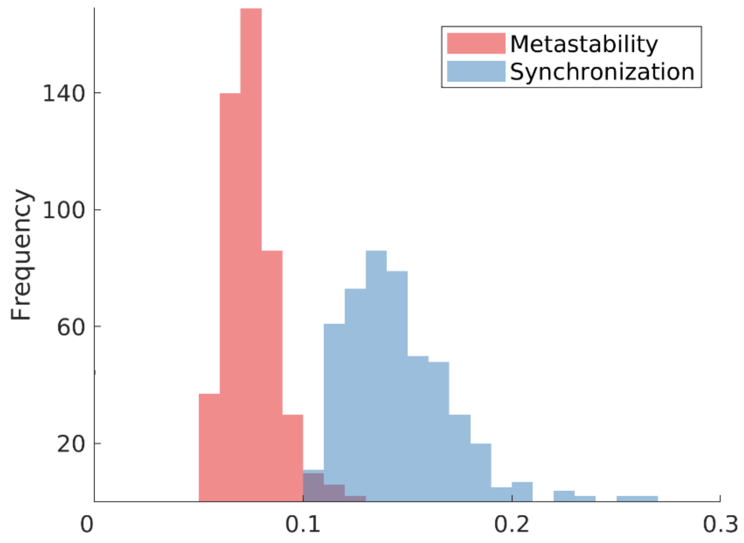

Supplementary Figure 3. *Distribution of global synchrony and global metastability as indicative of macroscopic coherence of the whole-brain network are illustrated for the empirical BOLD signals.*

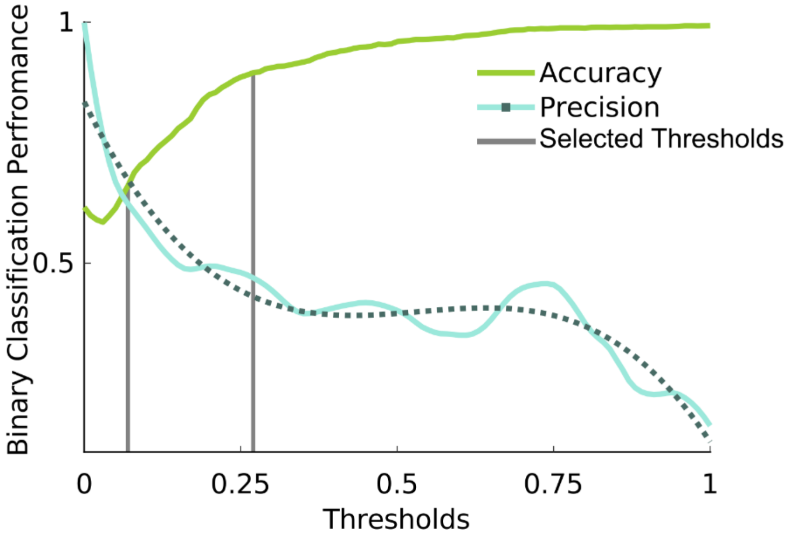

Supplementary Figure 4. *The accuracy and precision of the binary classification of edges at different thresholds ranging from 0 to 1 are illustrated. Cubic polynomial curve fitted to the precision is depicted as the dotted green curve. The locations of the intersection of two performance measures, as well as the knee point for both curves refer to the thresholds of 0.08 and 0.27, respectively.*

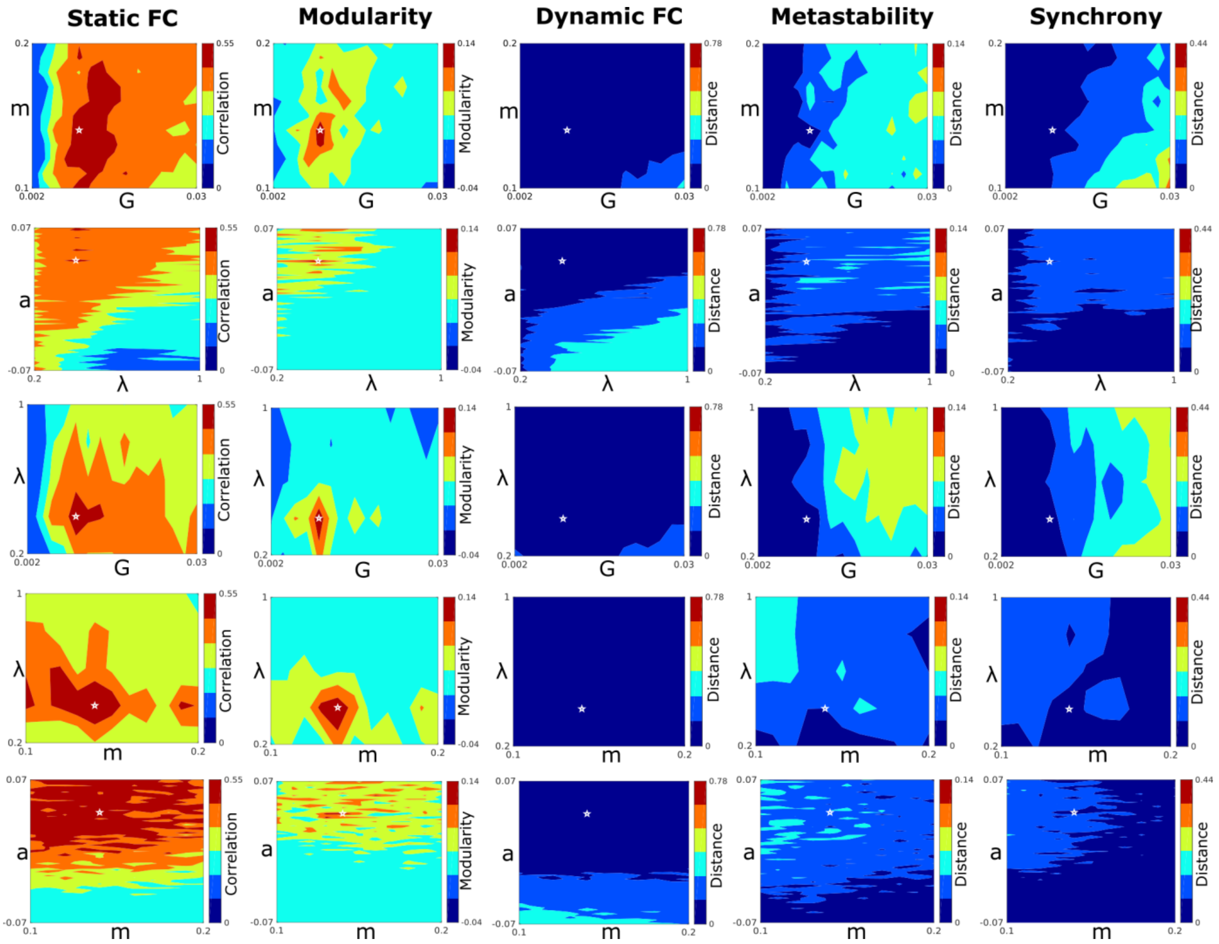

Supplementary Figure 5. *Parameter space search. This figure shows the exploration of the parameter space defined by the bifurcation parameter a, global coupling G, frequency lethargy λ and frequency modulation m. First column depicts the Pearson correlation between empirical and simulated static FC patterns for different pairings of parameters. Second column shows the whole brain modularity computed for the simulated static FC matrix. The Kolmogorov-Smirnoff distance between the similarities of coherence measures, obtained from the empirical BOLD data and simulated signals, as well as the difference of metastability and synchrony of simulated signals from the average metastability and synchrony measures of empirical BOLD signals are respectively illustrated in columns 3-5. Measures associated with the optimal choice of global coupling (G), bifurcation parameter (a), frequency lethargy λ and frequency modulation m are shown as a white asterisk (G=0.01, a=0.038, λ=0.4 and m=0.14).*

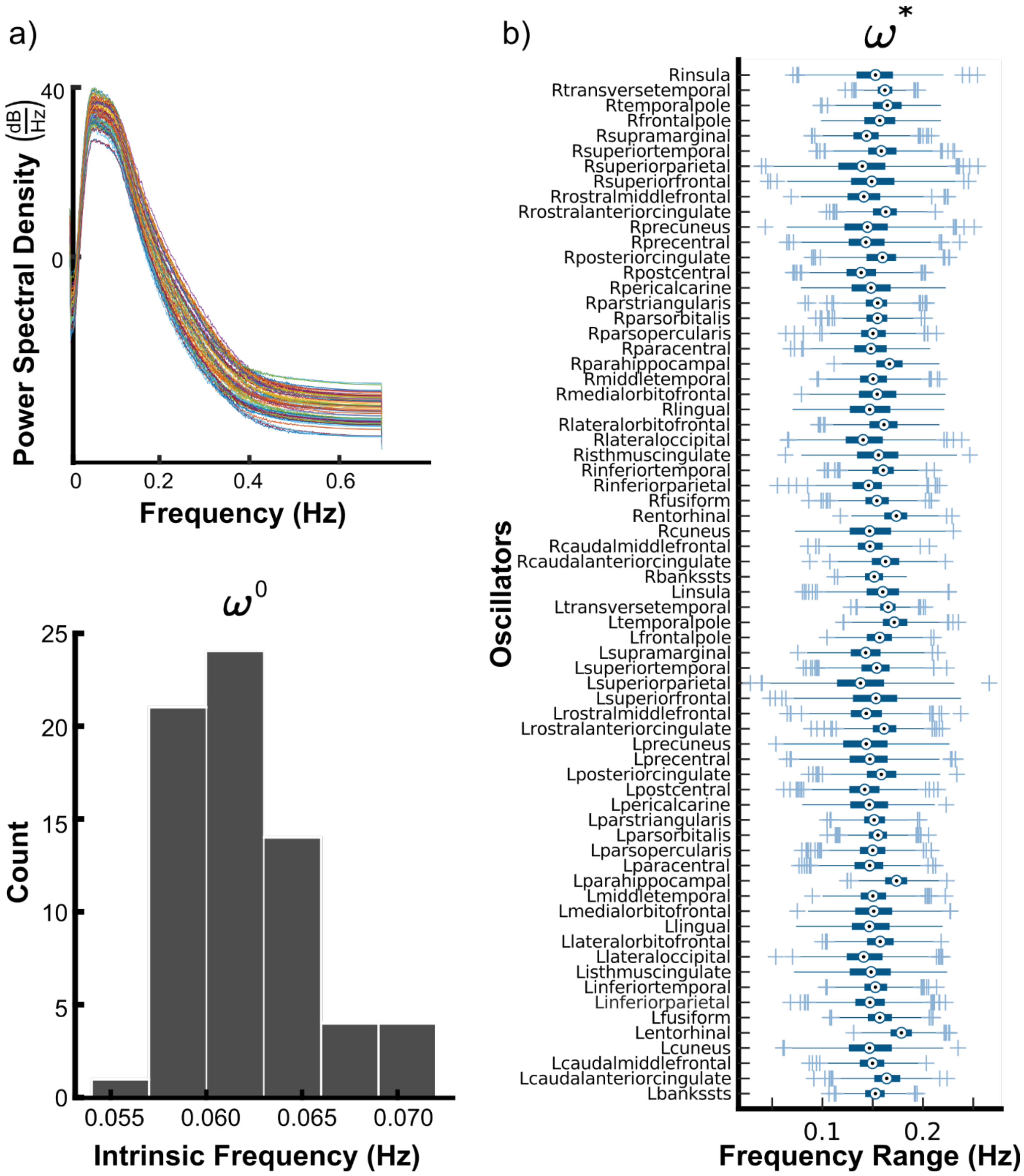

Supplementary Figure 6. *(a) The power spectral density and distribution of regional intrinsic frequencies, calculated as the median (across subjects) peak frequency of the regional BOLD signals. (b) The working frequency range of oscillators, centered at* $\boldsymbol{\omega}^* = \mathbf{2.5}\,\boldsymbol{\omega}^{\mathbf{0}}$.
